# Impact of a Mobile App–Based Health Coaching and Behavior Change Program on Participant Engagement and Weight Status of Overweight and Obese Children: Retrospective Cohort Study

**DOI:** 10.2196/14458

**Published:** 2019-11-15

**Authors:** Victor Cueto, C Jason Wang, Lee Michael Sanders

**Affiliations:** 1 Division of General Pediatrics Department of Pediatrics Stanford University School of Medicine Stanford, CA United States; 2 Division of General Internal Medicine Department of Medicine Rutgers New Jersey Medical School Newark, NJ United States; 3 Center for Policy, Outcomes, and Prevention Stanford University Stanford, CA United States

**Keywords:** child obesity, mHealth, mobile apps, health coaching, health behavior, self-monitoring, behavior change

## Abstract

**Background:**

Effective treatment of obesity in children and adolescents traditionally requires frequent in-person contact, and it is often limited by low participant engagement. Mobile health tools may offer alternative models that enhance participant engagement.

**Objective:**

The aim of this study was to assess child engagement over time, with a mobile app–based health coaching and behavior change program for weight management, and to examine the association between engagement and change in weight status.

**Methods:**

This was a retrospective cohort study of user data from *Kurbo*, a commercial program that provides weekly individual coaching via video chat and supports self-monitoring of health behaviors through a mobile app. Study participants included users of *Kurbo* between March 2015 and March 2017, who were 5 to 18 years old and who were overweight or obese (body mass index; BMI ≥ 85th percentile or ≥ 95th percentile) at baseline. The primary outcome, engagement, was defined as the total number of health coaching sessions received. The secondary outcome was change in weight status, defined as the change in BMI as a percentage of the 95th percentile (%BMIp95). Analyses of outcome measures were compared across three initial commitment period groups: 4 weeks, 12 to 16 weeks, or 24 weeks. Multivariable linear regression models were constructed to adjust outcomes for the independent variables of sex, age group (5-11 years, 12-14 years, and 15-18 years), and commitment period. A sensitivity analysis was conducted, excluding a subset of participants involuntarily assigned to the 12- to 16-week commitment period by an employer or health plan.

**Results:**

A total of 1120 participants were included in analyses. At baseline, participants had a mean age of 12 years (SD 2.5), mean BMI percentile of 96.6 (SD 3.1), mean %BMIp95 of 114.5 (SD 16.5), and they were predominantly female 68.04% (762/1120). Participant distribution across commitment periods was 26.07% (292/1120) for 4 weeks, 61.61% (690/1120) for 12-16 weeks, and 12.32% (138/1120) for 24 weeks. The median coaching sessions (interquartile range) received were 8 (3-16) for the 4-week group, 9 (5-12) for the 12- to 16-week group, and 19 (11-25) for the 24-week group (*P*<.001). Adjusted for sex and age group, participants in the 4- and 12-week groups participated in –8.03 (95% CI –10.19 to –5.87) and –9.34 (95% CI –11.31 to –7.39) fewer coaching sessions, compared with those in the 24-week group (*P*<.001). Adjusted for commitment period, sex, and age group, the overall mean change in %BMIp95 was –0.21 (95% CI –0.25 to –0.17) per additional coaching session (*P*<.001).

**Conclusions:**

Among overweight and obese children using a mobile app–based health coaching and behavior change program, increased engagement was associated with longer voluntary commitment periods, and increased number of coaching sessions was associated with decreased weight status.

## Introduction

### Background

A total of 1 in 3 children in the United States is either overweight or obese [1,2]. Obese children and adolescents are at risk for carrying their excess weight into adulthood and developing multiple comorbidities, including diabetes and coronary disease as adults [3,4]. Clinical management guidelines and treatment algorithms call for a staged approach to the treatment of overweight and obese children, which aims to promote healthy lifestyle changes through behavioral counseling [4,5]. Effective behavioral interventions for pediatric obesity involve multiple components focused on promoting healthy eating and exercise habits [6]. The most effective interventions involve supporting both children and parents to set goals, incorporate stimulus control, utilize problem solving, and participate in self-monitoring while working to achieve behavior changes [6]. The US Preventive Services Task Force (USPSTF) recommends primary providers to either provide or refer children with obesity to comprehensive intensive behavioral interventions aimed at decreasing excess weight and improving overall weight status [6]. However, child participation in comprehensive intensive behavioral interventions and clinical weight management programs is often low, with considerably high program attrition [7-9]. Common barriers to participation in weight management programs identified by families include concerns regarding affordability, inflexible scheduling, conflicts with other activities, time commitment, distance and transportation, and misalignment between expectations and program services [8,10,11]. Conversely, facilitators of participation for children and families may include tailored treatment plans and individualized health coaching [10-12]. Mobile health (mHealth) and telehealth technologies may provide a unique opportunity to overcome barriers to participation in obesity treatment by providing individualized interventions on a family’s timeline and in their home environments [13-15].

Among obesity treatment trials for adults, mHealth tools appear to successfully assist patients in managing comorbidities, such as diabetes, improve physical activity and dietary behaviors, and achieve meaningful weight loss [16-22]. mHealth interventions in children and adolescents have been found to be effective at improving health behaviors and health outcomes across a wide reach of conditions [23]. In obesity treatment trials for children, the effect of mHealth tools is less clear, as they have been primarily studied as a component of larger, multifaceted interventions [13,15,24,25]. mHealth tools incorporated into obesity prevention and treatment trials for children vary in their ability to improve weight outcomes [15,24,25]. However, mobile technology has been shown to be well accepted, feasible, and effective at supporting self-monitoring and promoting changes of physical activity and dietary behaviors [13,24-26]. However, overall, there is limited evidence for the efficacy of mHealth interventions as stand-alone treatment modalities for pediatric obesity and weight management [13]. Although there are a number of commercially available mobile apps targeting weight-related physical activity and dietary behaviors in children, reviews of commercial apps have found that most lack high-quality information, include only a few behavior change techniques (BCTs), and are not rooted in evidence-based behavior change theories [27,28]. As a result, some have suggested the need to rigorously evaluate commercial and stand-alone mHealth interventions aimed at promoting health behavior change among overweight or obese children [13,27,28].

### Objectives

In this study, we aimed to assess the engagement of overweight or obese children with a commercially available mHealth tool (*Kurbo*), which provides individualized health coaching and self-monitoring support designed to improve diet and physical activity behaviors. The primary aim was to describe and compare the engagement of participants with health coaching sessions, as a condition of their commitment period. The secondary aim was to examine the association between coaching sessions received and the change in a participant’s weight status over time. We hypothesized that participants with longer commitment periods would engage in both more coaching sessions and have a trend toward greater weight loss.

## Methods

### Design

This was a retrospective cohort study of participants in a commercial, mobile app–based platform and program (*Kurbo*) designed to promote health behavior change and weight management through self-monitoring and health coaching support. The research team and investigators had no role in the development of the mobile app platform or the creation and delivery of program content.

### Program

**Figure 1:**
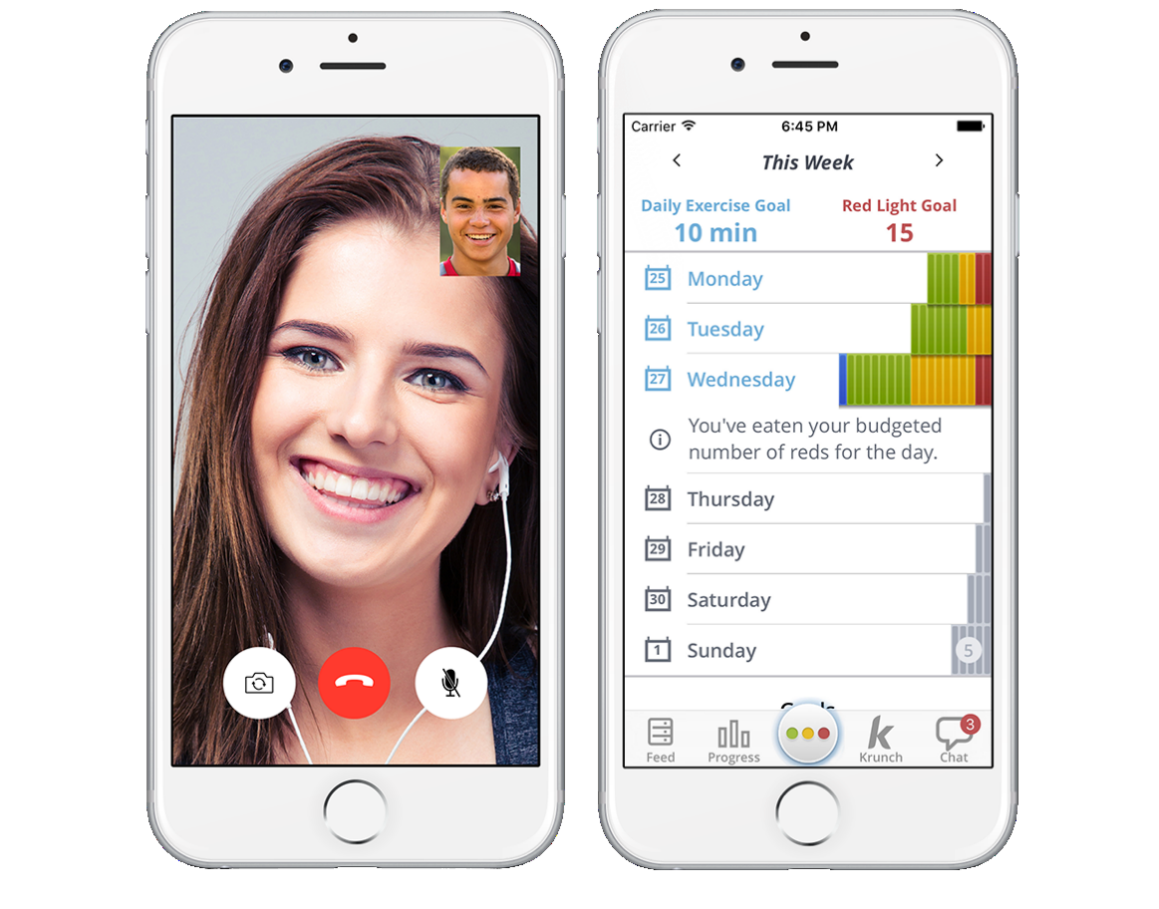
Mobile app platform.

The *Kurbo* mobile app platform (Figure 1) and program was designed to promote behavior change and encourage healthy lifestyle choices [29]. The program content and health coaching incorporate multiple BCTs, consistent with established taxonomy for behavior change interventions [30]. The BCTs emphasized in the program are linked to multiple theoretical frameworks, including the theory of reasoned action, theory of planned behavior, social cognitive theory, and control theory, as well as operant conditioning and the information motivation behavioral skills model [31].The program design was also informed by a model of supportive accountability, which emphasizes the essential role of human support in mHealth interventions [32]. The mobile app and program include 2 primary components: (1) self-monitoring of eating and physical activity behaviors through a mobile app interface and (2) individualized coaching sessions through video chat.

The self-monitoring component of the program employs the BCTs of self-monitoring of behavior and monitoring of outcomes of behavior [30]. Participants are encouraged to use the mobile app to log their daily food intake, using food categories adapted from the evidence-based traffic light system [33,34]. The traffic light system categorizes foods into 3 groups: unrestricted healthy green-light foods, less healthy yellow-light foods that should be eaten with caution, and unhealthy red-light foods that should be avoided [34]. The aim of this approach to eating behaviors is to encourage participants to gradually increase consumption of healthy foods (green lights) and decrease unhealthy foods (red lights) over time. This approach incorporates behavior substitution and habit formation [30]. Participants are also asked to self-monitor their physical activity behavior by logging the duration of activities in the mobile app, while working toward a goal of 60 min of moderate-to-vigorous physical activity each day.

The individualized coaching sessions component is provided by individuals who are hired and trained as coaches by *Kurbo*. Participants are paired with the same coach for the duration of their participation with the program, which aims to provide social support and accountability [30,32]. Coaches monitor the participant’s dietary and physical activity behaviors through a Web-based dashboard, which serves to reinforce self-monitoring and allows for feedback of behavior [30]. Each coaching session lasts about 15 min, and it is made available on a weekly basis. Coaching sessions emphasize review of past behavior and outcome goals, as well as support future outcome and behavior goal setting [30]. Coaches encourage self-talk and identification of behavioral cues, as well as assist with tailored problem solving and action planning [30]. For example, coaches may discuss environmental cues and identification of triggers to eat red-light foods while supporting goal setting and action planning for choosing more green-light foods. Additional topics addressed during coaching sessions may include understanding food labels and portion sizes. After each coaching session, participants receive an email from their coach, with praise for goals met and a tailored plan regarding goals set for the next week. Of note, parents are strongly encouraged to participate in coaching sessions; however, the program is not prescriptive regarding parental involvement. Similarly, the program allows for individually tailored coaching, but it is not specifically designed to address family dynamics or the age of participant.

In addition to coaching sessions, participants are also able to contact their coach between coaching sessions, via short message service text messages, email, or in-app messaging. Independent of the *Kurbo* mobile app platform, participants also have access to supplementary resources, including an emailed e-workbook, biweekly email newsletter, physical activity demonstration videos, blog posts, and downloadable healthy-eating cookbooks. These supplementary resources highlight BCTs by providing instructions on how to perform behaviors, support restructuring of the physical environment, and allow for social comparison. Finally, the program also makes use of the BCT of a behavioral contract [30].

### Participants and Data Source

The study examined a retrospectively identified cohort of participants who initially utilized the *Kurbo* mobile app and program between March 15, 2015 and March 15, 2017. Participants were not recruited for the purpose of conducting the study. Deidentified participant data from the *Kurbo* data registry were provided to the investigators for the purpose of the study. The study received an exemption from the Stanford University School of Medicine Institutional Review Board. All data were independently reviewed and inspected by the research team to confirm that the predetermined inclusion and exclusion criteria were met before analysis.

#### Inclusion Criteria

**Figure 2:**
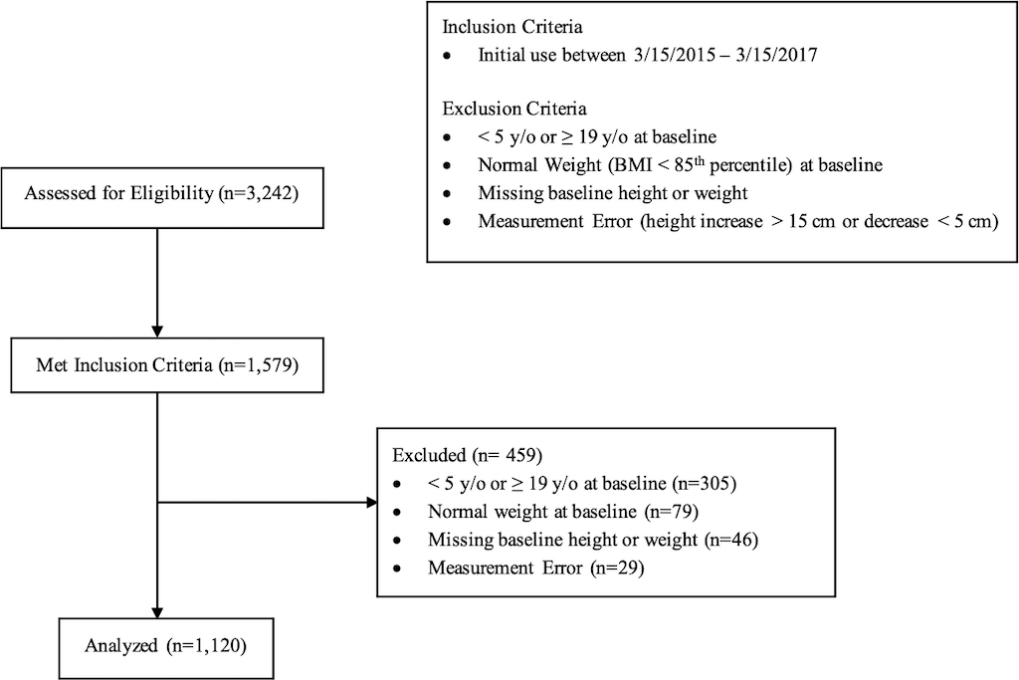
Cohort flow diagram.

The inclusion criterion (Figure 2) applied was participation during a defined 2-year period from March 15, 2015, to March 15, 2017.

#### Exclusion Criteria

The exclusion criteria (Figure 2) included age less than 5 years or age greater than or equal to 19 years upon initial use of the program. Additional exclusion criteria included a normal weight status (body mass index; BMI<85^th^ percentile) at baseline, as well as any data measurement errors, including missing baseline height, missing baseline weight, or any height velocity measurements exceeding 15 cm increase or 5 cm decrease (on the basis of established height velocity reference values) [35].

### Commitment Periods

Study participants were voluntarily subscribed or assigned to 1 of 3 commitment periods: 4 weeks, 12 to 16 weeks, or 24 weeks. Each participant was supported by either an employer-benefited plan, a health insurer–benefited plan, or a self-paid plan. The cost of the program is covered by either the parent (self-pay), a parent’s employer, or a family health insurance plan. Self-pay rates are dependent on the commitment period. Participants in self-paid plans voluntarily chose from 1 of 3 commitment periods: 4 weeks, 12 weeks, or 24 weeks. Those in employer- or health insurer–benefited plans were contractually assigned to 1 of 2 commitment periods: 12 weeks or 16 weeks. Of note, there were only 16 participants in the 16-week commitment period, and for analytic purposes, these participants were combined with the 12-week period to form a 12- to 16-week commitment period group. All participants had the ability to renew or change their plan at the end of the initial commitment period; however, data regarding renewals or changes were not available for analysis.

### Measures

#### Baseline Characteristics

Participant demographic characteristics were limited to self-reported age and sex, provided by either child or parent. Age in years at baseline was used to create 4 distinct age group categories. The age group categories were defined as 5 to 11, 12 to 14, and 15 to 18 years old. These age groups were informed by commonly reported groupings from population prevalence and large intervention studies [2,36]. The data registry objectively captured the payment source used for the program. Socioeconomic data, such as race, ethnicity, family income, or education were not reported. Self-reported baseline weight and height measurements were combined with age and sex, to calculate and derive the corresponding age and sex-specific BMI, BMI percentiles, and BMI expressed as a percentage of the 95^th^ BMI percentile (%BMIp95) at baseline. This was accomplished using SAS code, developed by the Centers for Disease Control and Prevention (CDC) for this purpose. All participants’ baseline weight was categorized according to CDC criteria, on the basis of age and sex-specific BMI percentile thresholds for children and adolescents. According to these criteria, weight status was defined as either overweight (≥85^th^ to <95^th^ BMI percentile) or obese (≥95^th^ BMI percentile). Obese participants were also categorized according to %BMIp95, a measurement of relative BMI, which is recommended by the CDC for children and adolescents with severe obesity [37]. This recommendation is based on analyses that have shown BMI scores to be poorly reflective of adiposity in youth with very high BMI measures and severe obesity. The measure of %BMIp95 is a more reliable measure of adiposity among obese youth and recommended for studies with a significant proportion of severely obese (%BMIp95≥120) children or adolescents [38]. Obese participants were additionally categorized according to 3 distinct classes of obesity, that is, Class I to III obesity, which are reflective of cardiometabolic risk and commonly used in obesity prevalence studies [2,4,39]. Class I is defined as ≥95^th^ BMI percentile; Class II corresponds with %BMIp95 ≥120 to <140% or BMI ≥35, whichever is lower; and Class III applies to %BMIp95 ≥140 or BMI ≥40, whichever is lower.

#### Primary Outcome: Participant Engagement

The primary outcome and measure of participant engagement was *coaching sessions*, defined as the total cumulative number of individual coaching sessions received by a participant during the participation period.

Other measures of participant engagement included participation period, program retention, coaching messages, dietary events, and physical-activity events. *Participation period* was defined as the total number of weeks between when the participant signed up for the program and the last recorded interaction with the app. The last recorded interaction with the app included logging of dietary or physical activity, an in-app text message sent to a health coach, or a coaching session. *Program retention* was defined as having a total participation period that was greater or equal to the intended commitment period in weeks. *Coaching messages* was defined as the cumulative total number of individual in-app text messages sent by each participant to his/her assigned health coach during the participation period. *Dietary events* was defined as the cumulative total number of self-reported individual foods logged by a given participant. *Physical activity events* was defined as the cumulative total number of self-reported individual physical activities logged by each participant during the participation period. The data registry did not capture whether dietary or physical activity events were self-reported by the participant or a parent.

#### Secondary Outcome: Change in Weight Status

A secondary outcome of the study was change in the participant’s weight status, defined as the *change in %BMIp95* between the self-reported baseline and endpoint measurements recorded during the participation period. The baseline measurement of %BMIp95 was derived from the participant’s initial self-reported weight and height measurement entered into the mobile app. The endpoint measurement of %BMIp95 was derived from the participant’s last self-reported height and weight measurement. There was no predetermined time interval between baseline and endpoint measurements.

### Analysis

Descriptive statistics were used to compare baseline characteristics (age, age group, sex, BMI percentile, %BMIp95, weight category, obesity class, and payment source), primary outcome (participant engagement), and secondary outcome (change in weight status) across the 3 commitment periods. Categorical variables were expressed as absolute values and corresponding percentages. Normally distributed continuous variables are reported as a mean with standard deviation (SD). The continuous engagement measures analyzed had nonnormal distribution patterns, each of these are reported as a median with interquartile range (IQR). Differences of measures across commitment period groups were explored using Chi-square tests for categorical variables and analysis of variance for normally distributed continuous measures. Similarly, differences across commitment periods for nonparametric continuous measures were analyzed using Kruskal-Wallis tests. Significance of change in weight status within commitment periods was analyzed using paired two-tailed *t* tests.

Multivariable linear regression models were constructed to examine 2 sets of associations: (1) between the primary outcome (number of coaching sessions) and each commitment period (reference of 24-week period) and (2) between the primary outcome (number of coaching sessions) and the secondary outcome (change in %BMIp95). Each multivariable model included adjustment for significant baseline differences in age group and sex. A sensitivity analysis, excluding involuntary participants (ie, health plan or employer supported), was performed to isolate differences associated with voluntariness of commitment period (Multimedia Appendix 1). All analyses were conducted using SAS Institute Inc software (SAS University Edition/SAS Studio 3.71).

## Results

### Participants

Of the 3242 participants assessed for eligibility, 1579 met the inclusion criteria. Of those, 305 participants were excluded for being outside the age range, normal weight at baseline (79), missing baseline data (46), or data measurement error. This yielded a final analytic sample of 1120 study participants, displayed in (Figure 1).

### Baseline Characteristics

The baseline characteristics for the study sample by commitment period are displayed in (Table 1). Overall, 292 participants were in the 4-week commitment period, 690 participants were in the 12- to 16-week group, and 138 participants were in the 24-week commitment period. Mean age at baseline was 12 years (SD 2.5), and most participants 68.04% (762/1120) were female. There were no statistically significant differences across the 3 groups in age or sex. The majority of participants 76.61% (858/1120) were categorized as obese, with mean BMI percentile (SD) of 96.6 (3.1) and mean (SD) %BMIp95 of 114.5 (16.5). Children in the 24-week group were more likely to be classified as obese, when compared with those in the 4- and 12-week groups (118/138, 85.5% vs 218/292, 74.7% and 522/690, 75.7%, respectively, *P*=.03). The predominant payment source (743/1120, 66.34%) was self-pay. The distribution of baseline characteristics for the study sample by age group are displayed in Multimedia Appendix 1. Except for payment source, all baseline characteristics differed significantly across age groups.

**Table 1:** Baseline participant characteristics by commitment period.

Baseline characteristics		All periods (N=1120)	4 weeks (n=292)	12-16 weeks^a^ (n=690)	24 weeks (n=138)	*P* value
Age (years), mean (SD)		12.0 (2.5)	11.9 (2.2)	12.0 (2.7)	12.0 (2.4)	.89^b^
**Age group,** **n (%)**						.61^c^
	5-11 years	573 (51.16)	150 (51.4)	350 (50.7)	73 (52.9)	
	12-14 years	392 (35.00)	109 (37.3)	237 (34.4)	46 (33.3)	
	15-18 years	155 (13.84)	33 (11.3)	103 (14.9)	19 (13.8)	
**Sex, n (%)**						.20^c^
	Male	358 (31.96)	103 (35.3)	218 (31.6)	37 (26.8)	
	Female	762 (68.04)	189 (64.7)	472 (64.1)	101 (73.2)	
Body mass index percentile, mean (SD)		96.6 (3.1)	96.4 (3.3)	96.5 (3.1)	97.3 (2.5)	.009^b^
%BMIp95^d^, mean (SD)		114.5 (16.5)	113.4 (17.5)	114.1 (19.3)	118.8 (19.3)	.01^b^
**Weight category^e^, n (%)**						.03^c^
	Overweight	262 (23.39)	74 (25.3)	168 (24.4)	20 (14.5)	
	Obese	858 (76.61)	218 (74.7)	522 (75.7)	118 (85.5)	
**Obesity class^f^, n (%)**						.39^c^
	Class I	508 (59.21)	128 (58.7)	315 (60.3)	65 (55.1)	
	Class II	236 (27.51)	65 (29.8)	140 (26.8)	31 (26.3)	
	Class III	114 (13.29)	25 (11.5)	67 (12.8)	22 (18.6)	
**Payment source^g^, n (%)**						<.001^c^
	Self-pay	743 (66.34)	292 (100)	314 (45.5)	138 (100)	
	Health plan	278 (24.82)	—^h^	278 (40.3)	—	
	Employer	99 (8.84)	—	99 (14.3)	—	

^a^12 to 16 weeks includes n=674 participants with 12-week and n=16 participants with 16-week commitment periods.

^b^Analysis of variance.

^c^Chi-square test.

^d^%BMIp95: percentage of the 95th BMI percentile.

^e^Categories by Centers for Disease Control and Prevention body mass index percentile for Age and Sex. Overweight (BMI Percentile ≥85 and <95th), Obese (BMI Percentile ≥95th).

^f^Obesity Class I (≥95^th^ to <120 %BMIp95), Class II (≥120 to <140 %BMIp95, or BMI ≥35), Class III (≥140 %BMIp95, or BMI ≥40), inclusive of N 858 participants categorized as obese.

^g^The 4 weeks and 24 weeks commitment periods consisted entirely of Self-pay participants, accordingly data for Health plan and Employer are not applicable.

^h^Not applicable.

### Participant Engagement

The engagement of participants with the mobile app program, compared across commitment periods, is displayed in (Table 2).

The primary outcome of median number of coaching sessions received was 8 (IQR 3-15) for the 4-week group, 9 (IQR 5-12) for the 12- to 16-week group, and 19 (IQR 11-25) for the 24-week group (*P*<.001). Overall, the median number of coaching sessions per participant was 9, with an IQR of 5 to 15. The median (IQR) values for other engagement measures were as follows: participation period, 15 weeks (IQR 12-30); the number of coaching messages, 3 (IQR 0-10); the number of logged dietary events, 174 (IQR 83-325); and the number of logged physical activity events, 42 (IQR 15-91). Median weeks of participation differed across commitment periods: 16 weeks (IQR 8-36) for the 4-week group, 14 weeks (IQR 12-22) for the 12- to 16-week group, and 30 weeks (IQR 22-51) for the 24-week group. Overall, program retention was high, with 79.91% (895/1120) of participants remaining engaged with the program for at least the duration of their commitment period. Program retention across commitment periods was 92.5% (270/292) for the 4-week group, 76.8% (530/690) for the 12- to 16-week group, and 68.8% (95/138) for the 24-week group (*P*<.001). In addition, the engagement of all participants stratified by age group is shown in (Table 3). There were no statistically significant differences in engagement by age group.

**Table 2:** Engagement of participants with mobile app–based program by commitment period, among all participants (N=1120).

Engagement measures	All periods (N=1120)	4 weeks (n=292)	12-16 weeks^a^ (n=690)	24 weeks (n=138)	*P* value
Coaching sessions^b^, median (IQR^c^)	9 (5-15)	8 (3-16)	9 (5-12)	19 (11-25)	<.001^d^
Coaching messages^e^, median (IQR)	3 (0-10)	4 (0-11)	3 (0-9)	6 (1-14)	<.001^d^
Dietary events^f^, median (IQR)	174 (83-325)	163 (80-321)	153 (76-278)	335 (188-596)	<.001^d^
Physical activity events^g^, median (IQR)	42 (15-91)	42 (15-98)	36 (13-78)	76 (33-152)	<.001^d^
Participation period^h^, median (IQR)	15 (12-30)	16 (8-36)	14 (12-22)	30 (22-51)	<.001^d^
Program retention^i^, n (%)	895 (79.91)	270 (92.5)	530 (76.8)	95 (68.8)	<.001^j^

^a^Includes 674 participants with 12-week and 16 participants with 16-week commitment periods.

^b^Median of total number of coaching sessions between participant and coach.

^c^IQR: interquartile range.

^d^Kruskal-Wallis Test.

^e^Median of total number of text messages from participant to coach.

^f^Median of total number of dietary event food logs recorded by participants (n=1100), otherwise missing.

^g^Median of total number of physical activity event logs recorded by participants (n=1078), otherwise missing.

^h^Median of total weeks between sign up and last recorded interaction with the app.

^i^Proportion of participants who completed equal or greater weeks than initial commitment period.

^j^Chi-square test.

**Table 3:** Engagement of participants with mobile app–based program by age group, among all participants (N=1120).

Engagement measures	All age groups, (N=1120)	5-11 years (n=573)	12-14 years (n=392)	15-18 years (n=155)	*P* value
Coaching sessions^a^, median (IQR^b^)	9 (5-15)	10 (5-15)	9 (5-14)	10 (6-15)	.77^c^
Coaching messages^d^, median (IQR)	3 (0-10)	3 (0-10)	3 (0-12)	3 (0-10)	.94^c^
Dietary events^e^, median (IQR)	174 (83-325)	171 (80-318)	175 (84-330)	177 (87-342)	.68^c^
Physical activity events^f^, median (IQR)	42 (15-91)	44 (15-89)	41 (15-92)	36 (13-102)	.89^c^
Participation period^g^, median (IQR)	15 (12-30)	15 (12-32)	15 (11-27)	14 (12-27)	.33^c^
Program retention^h^, n (%)	895 (79.91)	493(79.1)	314 (80.1)	128 (82.6)	.62^i^

^a^Median of total number of coaching sessions between participant and coach.

^b^IQR: interquartile range.

^c^Kruskal-Wallis Test.

^d^Median of total number of text messages from participant to coach.

^e^Median of total number of dietary event food logs recorded by participants (n=1100), otherwise missing.

^f^Median of total number of physical activity event logs recorded by participants (n=1078), otherwise missing.

^g^Median of total weeks between sign-up and last recorded interaction with the app.

^h^Proportion of participants who completed equal or greater weeks than initial commitment period.

^i^Chi-square test.

Results of unadjusted and adjusted models for the primary outcome (number of coaching sessions per participant) are displayed in Table 4. After adjustment for child sex and age group (with the 24-week group as reference), the 12- to 16-week group was associated with the fewest number of coaching sessions per participant, with a beta-coefficient of –9.34 (95% CI –11.30 to –7.39), in contrast to the 4-week group, with a beta-coefficient of –8.03 (95% CI –10.19 to –5.87). The results of sensitivity analyses restricted to only self-pay (voluntary) participants are included in Multimedia Appendix 1. In this subpopulation, the 4-week group was associated with the fewest number of coaching sessions per participant, with a beta-coefficient of –8.06 (95% CI –10.56 to –5.56), in contrast to the 12-to 16-week group, with a beta-coefficient of –6.02 (95% CI –8.48 to –3.56).

**Table 4:** Factors associated with total number of coaching sessions, among all participants (N=1120).

Participant factors		Unadjusted^a^ beta-coefficient (95% CI)	*P* value	Adjusted^b^ beta-coefficient (95% CI)	*P* value
**Age group (years)**					
	Age 5-11 years (reference: 15-18 years)	–0.17 (–2.14 to 1.79)	.86	–0.13 (–2.03 to 1.78)	.89
	Age 12-14 years (reference: 15-18 years)	–0.59 (–2.68 to 1.44)	.56	–0.53 (–2.53 to 1.46)	.59
**Sex**					
	Male (reference: female)	–1.38 (–2.77 to 0.01)	.05	–1.15 (–2.50 to 0.19)	.09
**Commitment period**					
	4 weeks (reference: 24 weeks)	–8.15 (–10.31 to –5.98)	<.001	–8.03 (–10.19 to –5.87)	<.001
	12-16 weeks (reference: 24 weeks)	–9.41 (–11.36 to –7.46)	<.001	–9.34 (–11.30 to –7.39)	<.001

^a^Unadjusted bivariate linear regression model of coaching sessions outcome as a function of age group, sex, or commitment period.

^b^Adjusted multivariable linear regression model of coaching sessions outcome adjusted as a function of age group, sex, and commitment period.

### Change in Weight Status

Within each commitment period, the mean change between baseline and endpoint for %BMIp95 was –5.4 (95% CI –6.2 to –4.5) for 4 weeks (*P*<.001), –4.8 (95% CI –5.3 to –4.3) for 12 to 16 weeks (*P*<.001), and –6.9 (95% CI –8.3 to –5.6) for 24 weeks (*P*<.001). Compared across age groups, the mean change of %BMIp95 was –5.6 (SD 7.9) for 5 to 11 year olds, –4.7 (SD 5.9) for 12 to 14 year olds, and –5.2 (SD 5.6) for 15 to 18 year olds (*P*=.09). Adjusting for age group and sex within each commitment period, the beta-coefficient per coaching session was –0.25 (95% CI –0.32 to –0.18) for the 4-week group, –0.16 (95% CI –0.21 to –0.11) for the 12-week group, and –0.26 (95% CI –0.34 to –0.18) for the 24-week group (*P*<.001). The multivariable model of all participants, adjusted for initial commitment period, age group, and sex, demonstrated an overall beta-coefficient decrease of –0.21 (95% CI –0.25 to –0.17) in %BMIp95 per each coaching session received (*P*<.001).

## Discussion

### Principal Findings

This retrospective study described the engagement of a large cohort of children and adolescents, with a multicomponent mobile app–based comprehensive behavioral program aimed at promoting healthy dietary and exercise lifestyle behaviors. Unlike traditional behavioral interventions and clinical weight management programs, which largely rely on in-person visits and sessions [6,40,41], mHealth programs, such as the one studied, enable participants to self-monitor health behaviors and receive health coaching at their own pace. As such, the findings of this study generally add to the growing evidence base for mHealth tools, more specifically for mobile app–based comprehensive behavioral programs to support health behavior change for overweight or obese children and adolescents.

Our findings of overall engagement with a median of 9 (IQR 5-15) coaching sessions during the participation period is notable for an mHealth program. This level of engagement, although considered low intensity by USPSTF criteria, is comparable with contact levels of in-person weight management programs [40,41]. We also documented consistent levels of participant engagement with other measures, such as self-monitoring of physical activity and dietary habits. The documented engagement of participants with both individualized coaching and self-monitoring is an important finding, given that these components represent behavioral change techniques that are known to be effective in managing pediatric obesity [42]. Furthermore, overall program retention with this mHealth program was high, and attrition was 20.9%, which is considerably lower than attrition rates of between 37% and 41%, reported for traditional in-person (non-mHealth) weight management programs [8].

Finally, in this observational study, we found a significant association between the number of coaching sessions and the change in self-reported weight status during participation in the program. This association suggests that greater exposure to coaching sessions in this study was correlated with increased weight reduction. USPSTF analyses of intensive in-person interventions suggest a dose-response relationship between intervention hours received and beneficial changes in weight, with effective programs requiring at least 26 contact hours [6]. Although this study design was unable to capture a participant’s absolute contact hours with the app or coaching sessions, the observed association between weight change and coaching sessions suggests a possible lower dose or lower threshold effect for this mHealth intervention, when compared with traditional in-person interventions. However, further research is necessary to validate the nature and magnitude of this association, as well as to rule out other explanations, including selection bias or reporter bias.

### Strengths and Limitations

The study has limitations common to other studies of digital health and mHealth interventions [15]. As an observational retrospective study design, the study also lacked an independent control or comparison group. The study is subject to some reporting bias, as all measures, including anthropometrics, were self-reported by participants. The study is also subject to selection bias, both because enrollment and participation were self-directed and because full functionality required access to an internet-connected mobile device. Another limitation was unmeasured confounders, including race/ethnicity, parental educational attainment, and household income, which limits adjustment and generalization, particularly for low-income populations [2]. However, the inclusion of employer-benefited and health plan–benefited participants, which may have included both commercial and government sponsored plans, likely increased the heterogeneity of the sample. Finally, the study as designed was unable to fully account for other factors that may influence intervention fidelity, including quality of coaching sessions, role of parents, clustering by coach, or use of other resources. Specifically, the study could not account for the degree of parental involvement and supervision among younger participants as compared with older participants. Nonetheless, the findings do not support a significant difference between age groups.

Still, rigorous and independent studies of digital health and mHealth interventions are limited, and this study meaningfully contributes to the literature on digital health and mHealth interventions focused on obesity treatment and prevention. First, the intervention was comprehensively designed with multiple components that have been shown to be effective strategies for weight management and behavior change in pediatric populations [6,30,33,34,42]. This is in contrast to the often limited BCTs employed in other mHealth tools [27,28]. Second, the intervention leveraged effective BCTs to support self-guided behavior change and utilized health coaches in a family context [24]. Third, the study population included a broad distribution of age groups, including preadolescent children, adolescents, and late adolescents. In fact, the age, sex, and weight characteristics of the study population are similar to that of traditional in-person medical weight management programs [40,41]. In addition to obese participants, the intervention also included overweight participants, which suggests generalizability of the findings beyond this study. Finally, it is notable that engagement with the program and change in weight status was consistent across age groups that include children, preadolescents, and adolescents.

### Implications

Our findings have implications for clinical care, population health, and public policy. Clinicians providing obesity treatment may consider the incorporation of mHealth programs, such as the one studied here, as an adjunct to clinic visits and traditional medical management strategies. Health care systems aiming to improve population health management efforts might find these types of mHealth solutions more accessible for providing access to care for patients in rural areas where availability of providers may be limited or to patients in urban areas, who may be restricted by long commute times or have limited transportation options. Finally, public health leaders and policy makers may be encouraged by the role that emerging digital technologies could play in addressing obesity at the community level.

### Conclusions

This study of a mobile app–based health behavior change and health coaching program among a large cohort of overweight and obese participants demonstrated high participant engagement. Increased engagement with coaching sessions was associated with longer voluntary commitment periods. Overall program retention was higher than that reported for similar in-person intensive behavioral interventions and weight management programs. Participant engagement with coaching sessions was associated with decreases in weight status (%BMIp95). Taken together, these findings highlight the potential of mHealth platforms as a promising model for delivering behavioral interventions that support weight management and behavior change for overweight or obese children and adolescents.

## Appendix

Multimedia Appendix 1 Supplementary tables – Baseline participant characteristics by age group, all participants (N=1120) and Factors associated with total number of coaching sessions, among self-pay (voluntary) participants (N=743).

## Abbreviations

%BMIp95: BMI as percentage of the 95th percentile
BCT: behavior change technique
BMI: body mass index
CDC: Centers for Disease Control and Prevention
CHRI: Child Health Research Institute
HRSA: Health Resources and Services Administration
IQR: interquartile range
LPCH: Lucile Packard Children’s Hospital
mHealth: mobile health
USPSTF: US Preventive Services Task Force
