# Supplementary material for: Impact of a Mobile App–Based Health Coaching and Behavior Change Program on Participant Engagement and Weight Status of Overweight and Obese Children: Retrospective Cohort Study
Source: JMIR Mhealth Uhealth. 2019 Nov 15;7(11):e14458. doi: 10.2196/14458 (PMC6884716; doi:10.2196/14458)
Supplement: Multimedia Appendix 1 [file mhealth_v7i11e14458_app1.pdf]

Multimedia Appendix I. Supplementary Tables

Table 5. Baseline participant characteristics by age group, all participants (N=1120).

Baseline characteristics		All groups, (N = 1120)	5-11 years, (n=573)	12-14 years, (n=392)	15-18 years, (n=155)	*P* value
**Age (years), mean (SD)**		12.0 (2.5)	9.9 (1.4)	13.3 (0.9)	16.3 (0.8)	<.001[a]
**Sex, n (%)**						.005[b]
	Male	358 (31.96)	201 (56.2)	124 (34.6)	33 (9.2)	
	Female	762 (68.04)	372 (48.8)	268 (35.2)	122 (16)	
**Body mass index percentile, mean (SD)**		96.6 (3.1)	97.0 (2.7)	95.9 (3.3)	96.7 (3.3)	<.001[a]
**%BMIp95[c], mean (SD)**		114.5 (16.5)	115.9 (17.9)	110.9 (18.6)	118.2 (21.4)	<.001[a]
**Weight category[d], n (%)**						<.001[b]
	Overweight	262 (23.39)	98 (17.1)	129 (32.9)	35 (22.6)	
	Obese	858 (76.61)	475 (82.9)	263 (67.1)	120 (77.4)	
**Obesity class[e], n (%)**						<.001[b]
	Class I	508 (59.21)	286 (60.2)	171 (65.0)	51 (42.50)	
	Class II	236 (27.51)	135 (28.4)	62 (23.6)	39 (32.50)	
	Class III	114 (13.29)	54 (11.4)	30 (11.4)	30 (25.0)	
**Payment source, n (%)**						.13[b]
	Self-pay	743 (66.34)	390 (68.1)	257 (65.6)	96 (61.9)	
	Health plan	278 (24.82)	126 (22.0)	108 (27.5)	44 (28.4)	
	Employer	99 (8.84)	57 (9.9)	27 (6.9)	15 (9.7)	

[a]Analysis of variance.
[b]Chi-square test.
[c]Percentage of the 95th BMI percentile.
[d]Categories by Centers for Disease Control and Prevention body mass index percentile for Age and Sex. Overweight (BMI Percentile ≥85 and <95th), Obese (BMI Percentile ≥95th).
[e]Obesity Class I (≥95th to <120 %BMIp95), Class II (≥120 to <140 %BMIp95, or BMI ≥35), Class III (≥140 %BMIp95, or BMI ≥40), inclusive of N 858 participants categorized as obese.

Table 6. Factors associated with total number of coaching sessions, among self-pay (voluntary) participants (N=743).

Participant factors	Unadjusted[a] beta-coefficient (95% CI)	*P* value	Adjusted[b] beta-coefficient (95% CI)	*P* value
**Age group (years)**				
Age 5-11 years (reference: 15-18 years)	-1.12 (-3.94 to 1.69)	.44	-0.88 (-3.64 to 1.88)	.53
Age 12-14 years (reference: 15-18 years)	-1.64 (-4.59 to 1.32)	.28	-1.25 (-4.15 to 1.65)	.40
**Sex**				
Male (reference: female)	-1.11 (-3.09 to 0.85)	.27	-0.66 (-2.60 to 1.28)	.51
**Commitment period**				
4 weeks (reference: 24 weeks)	-8.12 (-10.61 to -5.63)	<.001	-8.03 (-10.53 to -5.53)	<.001
12-16 weeks (reference: 24 weeks)	-6.02 (-8.48 to -3.56)	<.001	-6.02 (-8.49 to -3.56)	<.001

[a]Unadjusted bivariate linear regression model of coaching sessions outcome as a function of age group, sex, or commitment period.
[b]Adjusted multivariable linear regression model of coaching sessions outcome adjusted as a function of age group, sex, and commitment period.
